# Enhancing the in-plane shear properties of carbon fiber reinforced polymer composite laminates with aligned carbon nanofiber z-threads

**DOI:** 10.1007/s42114-026-01893-6

**Published:** 2026-06-02

**Authors:** Obaidul Hasan, Ryan A. Warren, Kuang-Ting Hsiao

**Affiliations:** 1 Department of Mechanical Engineering, University of South Alabama, Mobile, AL 36688 USA; 2 Systems Engineering Program, University of South Alabama, Mobile, AL 36688 USA

**Keywords:** Carbon fiber composites, Aligned carbon nanofibers, Z-threading, Advanced nanostructured composite, In-plane shear properties, Strain energy

## Abstract

In-plane shear strength plays a critical structural role of carbon fiber reinforced polymer (CFRP) laminates. However, the lack of reinforcement in the through-thickness direction (i.e., z-direction) makes a CFRP laminate susceptible to catastrophic failure when subjected to shear stresses between the angle plies. Previous studies reported that using interleaves of unaligned nano particles between the plies had negligible in-plane shear strength improvements. This study introduces a novel approach for improving in-plane shear properties through the use of carbon nanofibers (CNFs) z-threaded CFRP (ZT-CFRP), which utilizes electrically aligned CNFs as nano-reinforcement to thread through the gaps between carbon fibers along the z-direction and forms a mechanically interlocked multiscaled fiber reinforcement network. In-plane shear tests were conducted on [45/-45]_4s_ laminates of control CFRP, 0.5wt% unaligned CNF-modified CFRP, and ZT-CFRP samples containing 0.5 wt% and 1.0 wt% CNFs dispersed in the epoxy matrix. Compared with control CFRP, the 0.5wt% and 1.0wt% ZT-CFRP samples showed significant 25.6% and 22.2% increases in the in-plane shear strength and the modulus of toughness, respectively. While the control CFRP samples exhibited both intralaminar and interlaminar delamination failure modes, with damage spreading across the plies, the ZT-CFRP samples demonstrated a more localized failure confined to the interlaminar region, which indicated a significantly reinforced intralaminar region. In contrast, unaligned CNF-modified CFRPs were weaker and more brittle than both control CFRP and ZT-CFRPs in the testing values and microscopy. This study proved the importance of using advanced nanostructure arrangement when adding nanofibers inside CFRP laminates to improve the in-plane shear performance.

**Supplementary Information:**

The online version contains supplementary material available at 10.1007/s42114-026-01893-6.

## Introduction

### Background and previous studies

Recent advancements in nanomaterials and cutting-edge manufacturing technologies have significantly elevated the performance of carbon fiber and carbon fiber reinforced polymers (CFRP) composites. These innovations have allowed new levels of strength and performance to be achieved, making CFRPs a material of choice for industries demanding exceptional durability and lightweight characteristics. The primary objective of CFRP is to produce lightweight material with exceptional mechanical properties that cannot be achieved through the individual components alone [1]. The in-plane shear strength of carbon fiber reinforced polymer (CFRP) composite laminate is a major property that significantly influences the performance in various structural applications. In-plane shear properties are crucial because CFRP laminates are often subjected to complex loading conditions where forces are applied in multiple directions and angle ply lamination are needed; therefore, the ability to sustain the in-plane shear stress between the angle plies is critical to the laminate’s structural integrity.

CFRPs are anisotropic materials, meaning their mechanical properties vary depending on the direction being examined. Unlike metals, which are generally isotropic and have the same properties in all directions, the directional dependence of CFRP properties makes their behavior complex and require careful consideration in design and analysis, and sometimes, become weakness of CFRP structures. CFRP exhibits improved strength properties in the direction of fiber. Conversely, the strength capabilities are limited in the direction of through-thickness because of the absence of reinforcing fiber. Furthermore, when a laminate jointed by bolts or other similar approaches, the limited through-thickness strength could also cause the delamination failure of the structure. Guo et al. [2] studied that under low-velocity impact, composite laminates joined by bolts exhibit progressive damage dominated by matrix cracking, delamination and fiber failure. One of their key findings is that the stress distribution evolves in a characteristic “peanut-shaped” pattern aligned with the fiber direction. This indicates that the applied load travels preferentially along the direction of the reinforcing fibers rather than uniformly in all directions. Due to the anisotropy, delamination can easily occur when subjected to large interlaminar stresses. This phenomenon arises because the fibers within the plane of a laminate do not provide reinforcement along the through-thickness direction [3].

Various methods have been studied for z-directional (i.e., through-thickness directional) reinforcement to improve delamination resistance. The most common methods for z-directional reinforcement are unaligned nano particle reinforcement [4–7] and stitching [8, 9]. While fiber stitching enhances the inter-laminar properties of CFRPs, improving resistance against delamination and damage, it also potentially introduces challenges such as fiber damage, in-plane fiber displacement, increased manufacturing complexity, and reduced overall performance [9]. Therefore, a significant challenge in composite materials is the presence of weak interlaminar interfaces, which can lead to problems with reduced matrix-sensitive failure resistance and tolerance. These issues often result in the need for over-engineering, ultimately limiting the overall performance of composite structures. Using Z-pins is another way to improve strength in through thickness direction. Shi et al. [10] developed finite element model with continuum damage mechanics to analyze progressive damage in Z-pin reinforced composites. Their results show enhanced interlaminar performance due to Z-pins, while highlighting stress concentration and damage evolution near pin-resin regions. Despite the improved performance, Z-pins can reduce the in-plane mechanical properties, increase weight of the structure, introduce fiber waviness, create stress concentrations, lower fatigue performance, and increase manufacturing complexity in composite laminates [11, 12].

Reinforcement using nanoparticles is an effective approach to improve the material properties. The invention of carbon nanotubes has opened new possibilities, paving the way for groundbreaking advancements in nanomaterials [13, 14]. Carbon nanotubes (CNTs) and carbon nanofibers (CNFs) are among the most remarkable materials to emerge from the field of nanomaterials. Recently, the use of nanoparticles in reinforcement has become increasingly popular in research literatures [15]. An emerging focus in nanomaterials reinforcement research is the integration of nano-reinforced resin layers, often referred to as nanomaterials improved interlayers in Fiber Reinforced Polymer (FRP) laminates. This innovative approach significantly boosts the interlaminar strength, enhancing the overall performance and durability of CFRP [4, 5]. Nanofibers help in redistributing shear stress more efficiently, limiting the growth and occurrence of both cracks and delamination under loads. The presence of nanofibers enhances the CFRP matrix, improving interlaminar adhesion and increasing resistance to shear stress [16].

The application of nanomaterials can be categorized into two configurations: unaligned and aligned. McDonald et al. [17] compared the in-plane shear stress of control CFRP laminates and unaligned CNF/epoxy-interleaf-coated CFRP laminates. While the shear stresses in both samples were similar, their failure modes differed. The control samples showed interlaminar delamination spreading along their lengths, while the unaligned CNF-modified samples experienced localized failure. Kim [18] used CNT mats aiming at improving the in-plane shear strength, but no significant improvement was observed. A similar study was conducted by Fernández et al. [19] by using CNTs in glass fiber epoxy composite, but no significant improvement was observed. Kadir et al. [20] observed a slight improvement of in-plane shear strength after using unaligned CNTs as reinforcement. These studies [17–20] provide evidence that unaligned CNTs/CNFs have negligible effects in enhancing in-plane shear properties.

Another approach is using aligned nanofibers rather than just unaligned CNF or CNT in the polymer matrix. Ajayan et al. [21] first observed CNT alignment in polymer by applying shear force to polymer. Aligned nanofibers offer superior performance over unaligned CNFs in CFRP [22, 23]. Their alignment with the load direction improves load transfer, resulting in enhanced strength, stiffness, and fracture toughness [24–29]. Khan et al. [25] found that aligning CNTs significantly improves electrical and mechanical properties compared to randomly arranged CNTs. Preliminary toughening mechanisms in composites with aligned CNTs include crack tip deflection, bifurcation, and the bridging and pullout of individual and bundled CNTs. Villoria et al. [27] used aligned CNT forest between prepregs to increase in-plane strength related properties and achieved a 30% increase in bolt-bearing (i.e., tension-bearing) strength, a 14% boost in open-hole compression strength, and an over 25% enhancement in L-section bending energy. However, this system selectively uses the densely-packed self-vertically standing CNT forest between certain critical plies and portions of a laminate rather than through the whole laminate, potentially limiting their effectiveness in enhancing the overall strength, toughness, and performance of the entire laminate structure, especially if unexpected loads were applied significantly differently than originally designed loading scenarios.

Numerous studies have shown the significant impact of aligned nanomaterial reinforcement on enhancing material properties. For instance, Gupta et al. [30] demonstrated a remarkable 360% increase in electrical conductivity by utilizing aligned carbon nanotubes (CNTs), while Kirmse et al. [31] achieved an astounding 2840% improvement in the through-thickness electrical conductivity with 0.85 wt% carbon nanofibers (CNFs). These findings emphasize the role that nano reinforcement alignment technology plays in optimizing the performance of carbon fiber-reinforced polymers (CFRPs), offering considerable potential for advancing their applications in various industries.

### Research motivation

**Fig. 1:**
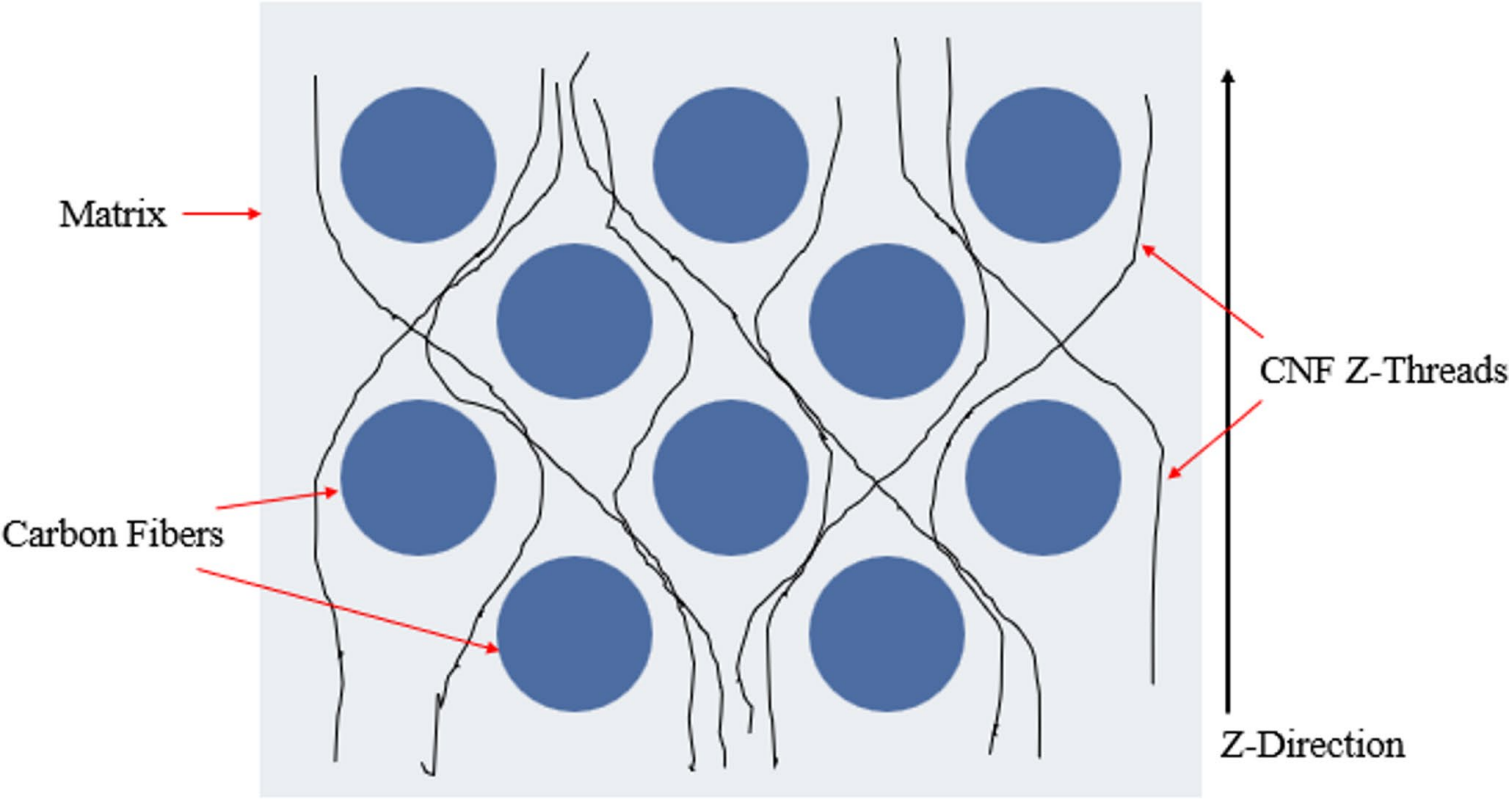
The principle behind ZT- CFRP

The previously mentioned studies suggest that the absence of reinforcement makes CFRP composites vulnerable to applied loadings along the thickness direction and CNFs/CNTs can be used as reinforcement medium. Also, the orientation of CNFs/CNTs has an impact on the role of nano materials when used for reinforcement. However, there is no literature to directly and explicitly report any significant improvement of using CNT or CNF for the CFRP laminates’ in-plane shear strengths yet, maybe due to individual type nanocomposites’ nano-reinforcing method difference, nano-reinforcing blind spots, or availability/affordability of large size testing samples. The study described in this paper will use a novel advanced nanostructured CFRP manufactured via a scalable roll-to-roll hot-melt prepreg process to investigate the roles of aligned CNFs on the in-plane shear strength and the modulus of toughness (i.e., strain energy absorption). The carbon nanofiber Z-Threaded CFRP (ZT-CFRP) manufacturing technique [32] aligns and threads long CNFs/CNTs between the carbon fibers along the z-direction (i.e., through-thickness direction) during the scalable roll-to-roll ZT-CFRP prepreg production process [33]. The long CNF z-threads reinforce layers by forming an interlocked multiscale fiber reinforcement network in both the interlaminar and intralaminar zones of the ZT-CFRP laminate. Figure 1 illustrates the multiscaled reinforcement architecture of the ZT-CFRP. Inside a tightly-packed carbon fiber bed, the carbon fibers are mechanically interlocked with long CNF-z threads which thread through the gaps between carbon fibers in a zigzag pattern and provide extra performance enhancement (e.g., through stress-sharing and energy dispersion) to the interlaminar and intralaminar zones in CFRP. The critical factor behind this ZT-CFRP architecture is that the diameter of the carbon fiber is about 5–7 μm and the CNF length is about 50–200 μm and based on the geometric augmentation; theoretically a long CNF could connect between 7 to 50 carbon fibers in an array of packed carbon fibers [23]. The network of multi-scale, interlocked nanostructured fiber-reinforcement arrays help enhance the matrix-sensitive properties of CFRP. The novel ZT-CFRP laminates have been experimentally proven to reliably and significantly improve various matrix-sensitive mechanical properties at room temperature [23, 31, 34], thermal conductivity [35], electrical conductivity [31], and the interlaminar shear load-bearing performance during extreme temperature exposure [28]. From the past performance reported by literatures and the unique nano-reinforcement architecture as shown in Fig. 1, the ZT-CFRP could have an impact on the composite laminate’s in-plane shear strength and the modulus of toughness.

Previous studies investigating the enhancement of in-plane shear properties through the incorporation of CNTs/CNFs interleaves have shown that the expected improvements were not significant. This suggests that the presence of unaligned nanofiber reinforcement between laminas does not contribute to improving the overall performance under the in-plane shear testing, limiting their effectiveness in enhancing the in-plane shear properties. On the other hand, the aligned nanofiber forest improved the in-plane shear related properties within the interlaminar zone of the selected area but there is no reinforcement inside each lamina of CFRP, ultimately limiting the whole laminate’s properties. This paper is to investigate the role of CNF z-threads in reinforcing the CFRP laminate, possibly in the interlaminar and intralaminar zones under the in-plane shear test. The in-plane shear properties will be characterized. The role of CNF concentration in the ZT-CFRP will be studied. The failure mechanisms and microstructural changes that occur after the fracture of ZT-CFRP laminates in comparison with traditional CFRP laminates will be observed. In this paper, unmodified control CFRP, 0.5 wt% Unaligned CNF-modified CFRP, 0.5 wt% and 1.0 wt% CNF-based ZT-CFRP were prepared, tested, and analyzed for finding more insights of the role of CNF z-threads to the in-plane shear properties of ZT-CFRP at room temperature.

## Experimentation

### Materials

The materials used in this experiment were: Hexcel AS7 unidirectional fabric (1.79 g/cm^3^ fiber density, 12k tow size, areal weight 190 g/m^2^), EPON 862 epoxy resin and Epikure-W curing agent (Miller-Stephenson Chemical Co. Inc.), CNF from Pyrograph Products Inc. (PR-24-XT-PS grade) which is reported to have a diameter of 100 nm and length from 20 to 200 μm, and S-191, S-192 surfactants from BYK.

### Hot-melt epoxy resin film preparation (for control CFRP prepreg)

During the preparation of control CFRP, Epon-862 epoxy resin was mixed with Epikure-W at a stochiometric ratio 100:26.5 and stirred manually. This solution was then kept within a vacuum chamber for 90 min at 120 °C for removing trapped air and B-staging the epoxy. A uniform, thin film was prepared with this B-staged epoxy using a proprietary hot-melt film-making process and machine. This hot-melt resin film was then cut into small pieces and individually stored in a freezer at −17 °C in preparation for the prepreg process.

### Hot-melt CNF/epoxy resin film preparation (for unaligned-CFRP prepreg and ZT-CFRP prepreg)

To make the unaligned CFRP, two concentrations of CNFs were used, i.e., 0.5 wt% and 1.0 wt% with respect to the total final mixture weight. For instance, 0.5wt.% CNFs were mixed within EPON-862 epoxy resin. Then 0.5wt.% of surfactants Disperbyk-191 and Disperbyk-192 were added and mixed using a high shear mixer. This mixture was then sonicated by a sonicator (QSONICA Q700) for 1 h and the dispersion was checked under a microscope to ensure no significant CNF aggregates. The solution was then kept within a vacuum chamber for 60 min at 120 °C for degassing the solution. After degassing the solution, Epikure-W was added maintaining stoichiometric ratio of 100:26.5 and stirred manually. The solution was kept in a degassing chamber for 90 min at 100 °C for B-staging. The B-staged thin film of unaligned CNF/epoxy for was prepared using a proprietary hot-melt film-making process and machine. For making the 1.0 wt% CNF/epoxy hot-melt resin film, all processing steps are the same except the concentration of CNFs, Disperbyk-191 and Disperbyk-192 were changed to 1.0 wt%.

### Roll-to-roll ZT-CFRP prepreg manufacturing (the same prepreg machine also used for control CFRP and unaligned-CFRP prepregs manufacturing)

**Fig. 2:**
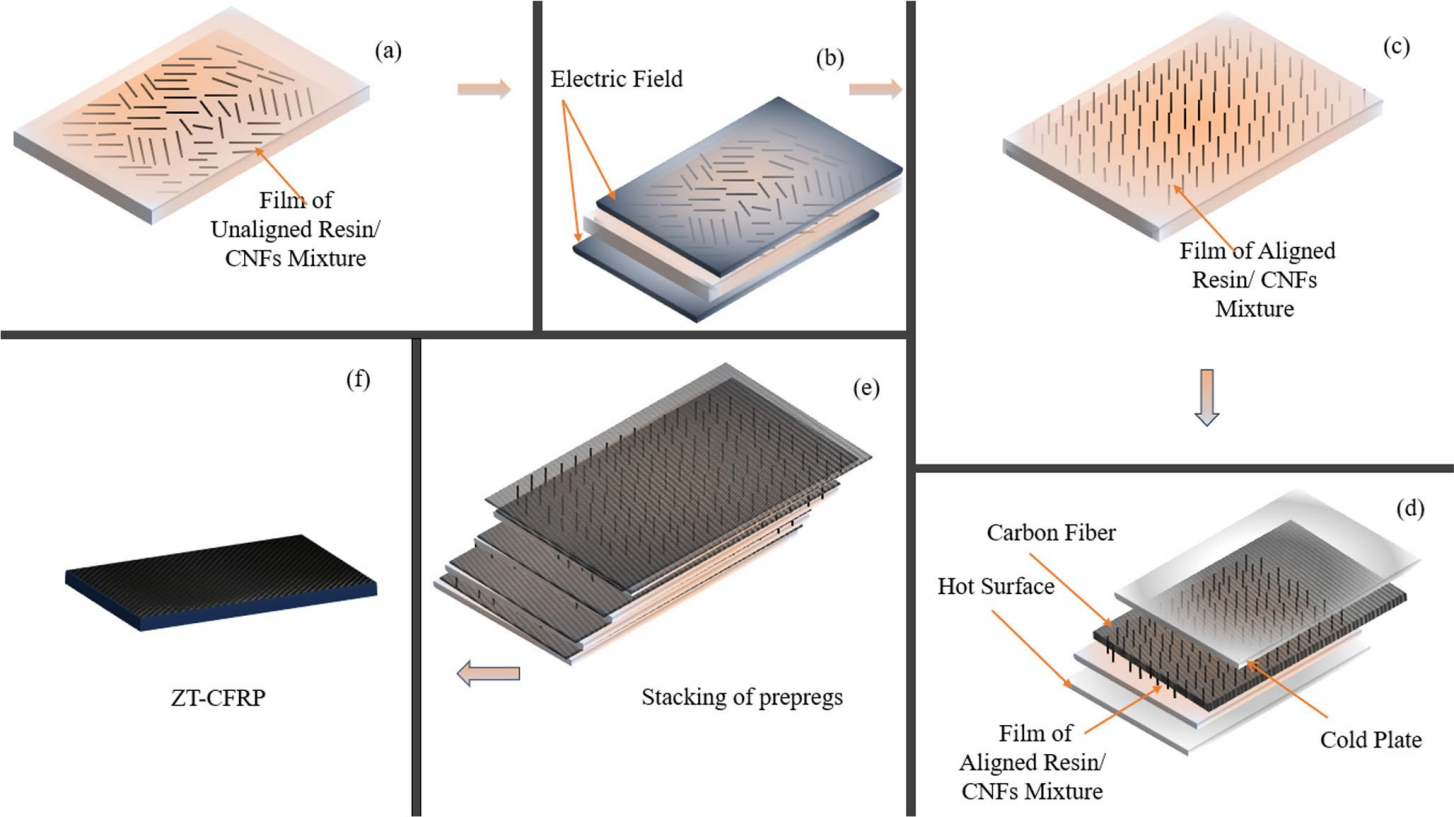
Depiction of the ZT-CFRP prepreg production processes (**a**) resin/CNFs mixture with Epikure-w (**b**) Unaligned CNFs under the electric field (**c**) aligned resin/CNFs mixture (**d**) aligned CNFs/resin mixture with carbon fiber in a prepreg manufacturing system (**e**) stacking of prepregs (**f**) ZT-CFRP

**Fig. 3:**
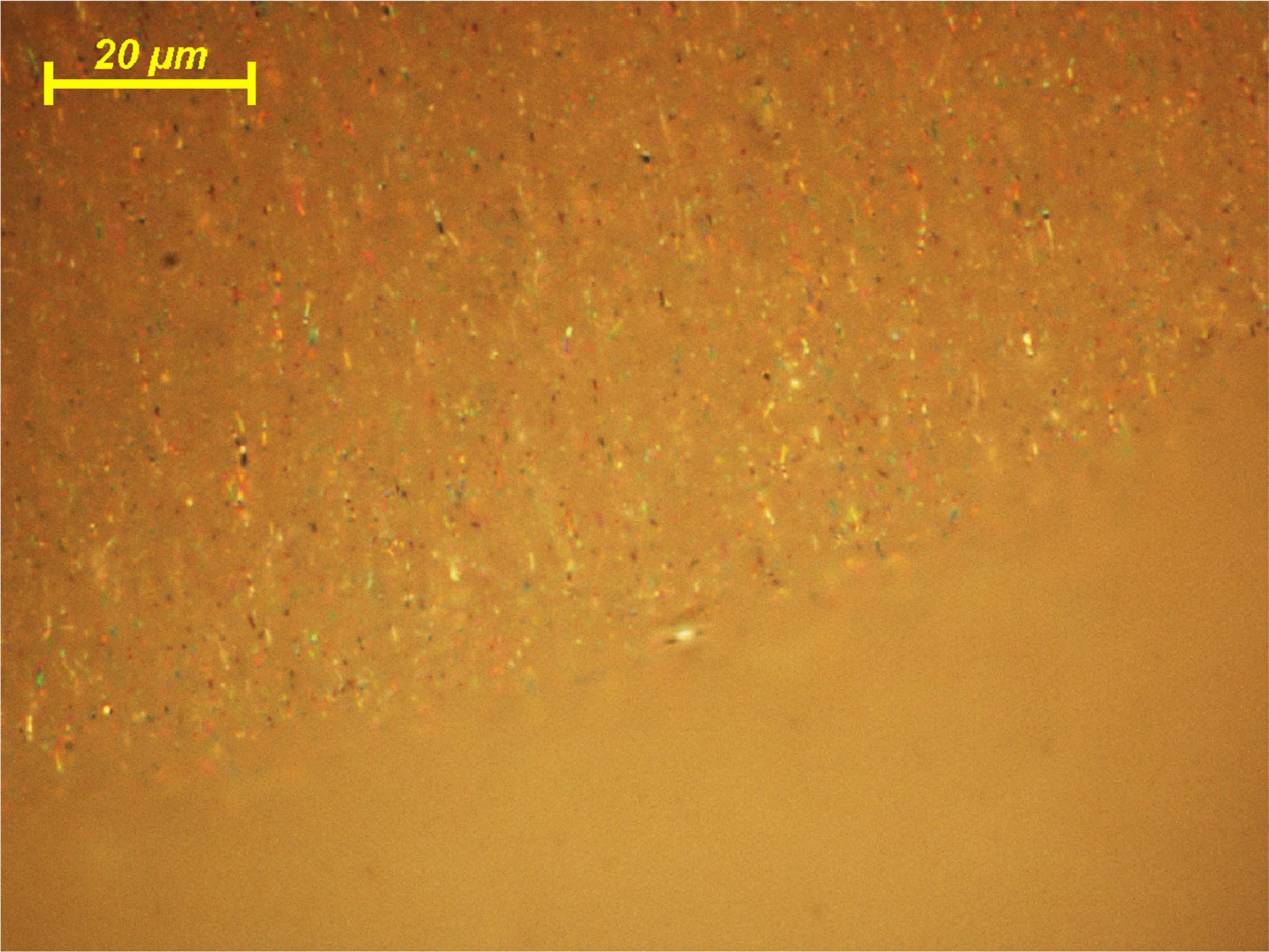
Microscopic image of the sideview of the electrical field induced z-alignment of CNFs inside a hot-melt CNF/epoxy film. Effective z-alignment of CNFs was accomplished by the electrical field

**Fig. 4:**
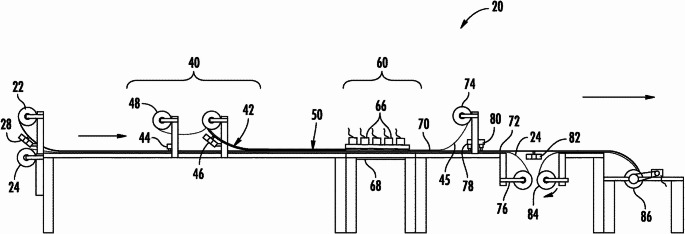
ZT-CFRP prepreg machine (US20240149541A1 [33]) for making ZT-CFRP prepreg (as well as for making control and unaligned CFRP prepreg under this study)

**Fig. 5:**
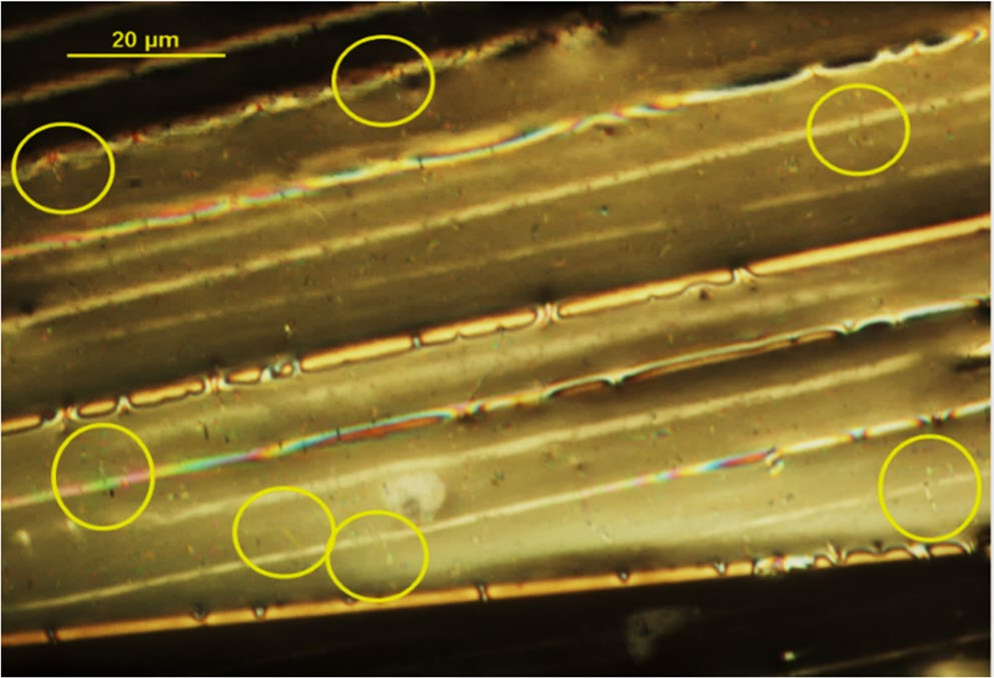
Microscope picture of sideview of a ZT-CFRP prepreg

The process principles to manufacture ZT-CFRP prepreg have been previously reported in [23, 32, 33, 35] and are illustrated in Fig. 2. The process started from Step (a), i.e., brought the freezer stored film of unaligned CNF/epoxy resin film to room temperature. Then, in Steps (b) and (c), the CNF/epoxy film was heated to 120 °C to melt the resin and was simultaneously subjected to a strong electrical field (~ 0.67 kV, 30 Hz) of waveform (of DC pulse, duty cycle: 50%) for ~ 90 s to quickly align the CNFs in the z-direction. Then the aligned CNF/epoxy film was quickly cooled by air to solidify the resin and lock the z-alignment of CNFs in the solid-phase resin film. The z-alignment of CNFs is critical to the success of ZT-CFRP prepreg production; a small piece of the hot-melt z-aligned CNF/resin film was observed under a microscope for quality check and confirm the z-alignment of the CNFs in the film before the next step. Figure 3 shows the microscope image of many CNFs aligned in the z-direction (i.e., perpendicular to the film surface edge) of a hot-melt CNF/epoxy film. Note that some CNFs were wavy due to the manufacturing process used by the CNF manufacturer, and it is not known yet if the waviness of long CNFs would affect the alignment quality due to limited options and data of long CNF products available in the market. In Step (d), The z-aligned CNF/resin film was placed on the proprietary (US20240149541A1 [33]) roll-to-roll ZT-CFRP prepreg machine (see Fig. 4). The ZT-CFRP prepreg machine used a cooling plate to press the z-aligned hot-melt CNF/resin film against a preheated carbon fiber fabric, which is heated and supported by a heating plate. When the hot-melt z-aligned CNF/resin film and dry carbon fabric pulled through the gap between the cooling plate and heating plate, as the resin touched the hot carbon fiber, the resin at the interface was locally melted instantly and allowed the z-aligned CNFs to gradually flow with the melted resin and being fed and threaded into the micro-scale cavities between carbon fibers and creating the desired ZT-CFRP microstructure as shown in Fig. 1. Once the CNF/epoxy resin film was completely pressed into the carbon fabric, air-cooling at room temperature was applied to solidify the B-staged resin and secure all carbon fibers and CNF z-threads and form the desired ZT-CFRP prepreg. At the output end of the ZT-CFRP prepreg machine, the ZT-CFRP prepreg was collected as a roll and wrapped with plastic wrap to be preserved in a freezer at −17 °C. Figure 5 showed the side-view of ZT-CFRP prepreg and one can see the surface-exposed portion of CNFs z-threaded perpendicular to carbon fibers. Unlike other methods described in the literature, ZT-CFRP prepreg manufacturing method is compatible with conventional hot-melt composite prepreg processing system with minimum amount of modification. And the ZT-CFRP prepreg is handled as a regular CFRP prepreg for the composite part manufacturing processes (e.g., vacuum bag process, autoclave curing process, and additive manufacturing) starting from the prepreg layup, degassing, molding, and curing steps. While other nanomaterial alignment strategies often require specialized handling and are typically limited to selective interface modification, the inherent compatibility of ZT-CFRP enables more straightforward integration into current production systems, highlighting its potential as a scalable solution for next generation nanostructured composite structures/parts manufacturing. The current automated roll-to-roll production speed in the lab scale of an up to 280 mm wide CFRP prepreg is approximately 300 mm per minute in the length of ZT-CFRP prepreg rolled out by the lab scale machine, which is sufficient to produce ZT-CFRP prepreg samples for many coupon-level tests; and further scaling up of the current ZT-CFRP prepreg production is possible by expanding the machine’s capacity and dimensions when need emerges.

To make a ZT-CFRP laminate, Steps (e) and (f) of Fig. 2 were applied, i.e., stacked the ZT-CFRP prepreg plies and manufactured them into a composite laminate. For making the control CFRP prepreg and unaligned CNF-CFRP prepreg for this study, the same roll-to-roll ZT-CFRP prepreg machine was used but the hot-melt pure resin films were not subjected to E-field Z-alignment process (i.e., Steps (b) & (c)).

### Out-of-autoclave-vacuum-bag-only process for composite laminate manufacturing

**Fig. 6:**
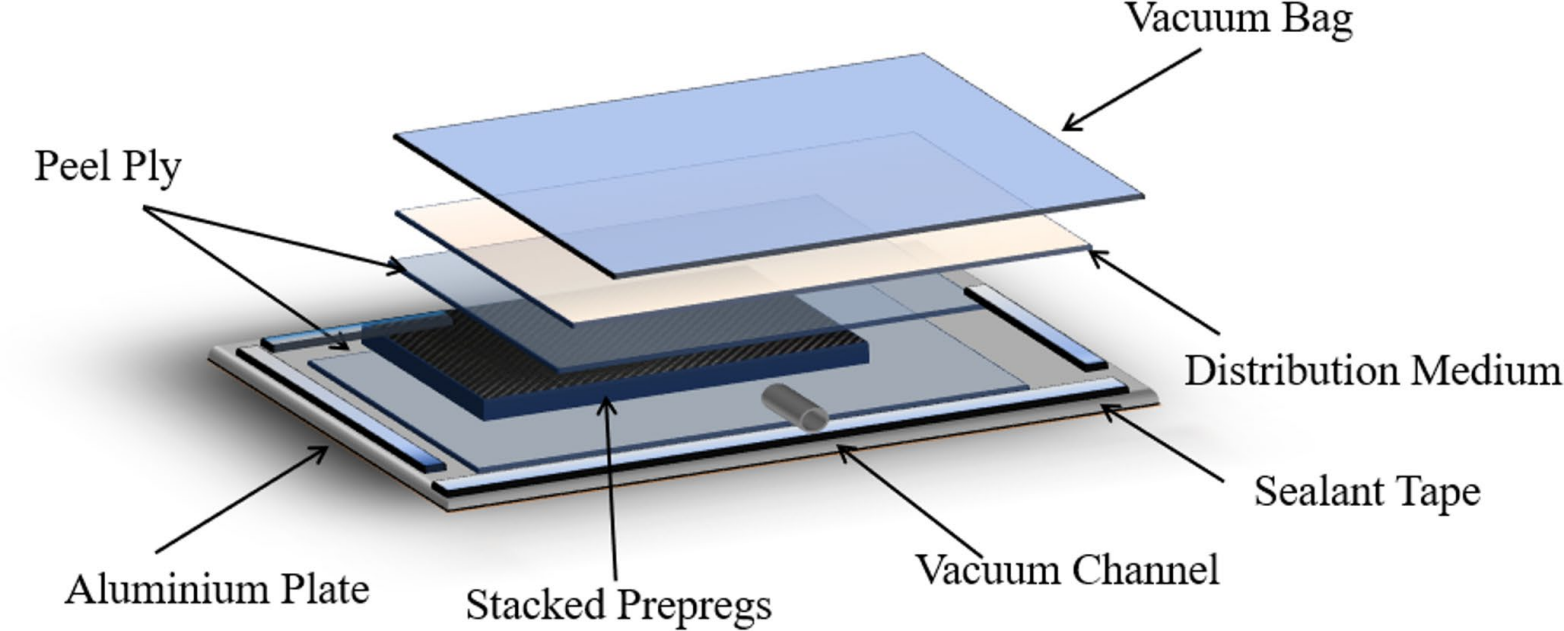
Schematic Diagram of OOA-VBO Setup

In this paper, Out-of-Autoclave-Vacuum-Bag-Only (OOA-VBO) method (as shown in Fig. 6) was used to manufacture composite laminates. Sixteen plies of 254.0 mm by 254.0 mm prepregs were stacked in a [45/−45]_4 S_ ply sequence in accordance with the ASTM D-3518 [36] standard and subjected to curing through the OOA-VBO method. In the OOA-VBO process, an aluminum plate was first coated with a mold release agent. The OOA-VBO used a bottom peel ply, the stacked CFRP plies, a top peel ply, a vacuum channel, sealant tape, and the vacuum bag, as depicted in Fig. 6. The entire setup was then placed in a hot press and cured for 120 min at 120 °C. After the curing cycle, the CFRP panel was allowed to cool within the vacuum bag. Following demolding, the cured panel underwent a post-curing process for 120 min at 180 °C to ensure the epoxy being fully cured.

### In-plane shear test

**Fig. 7:**
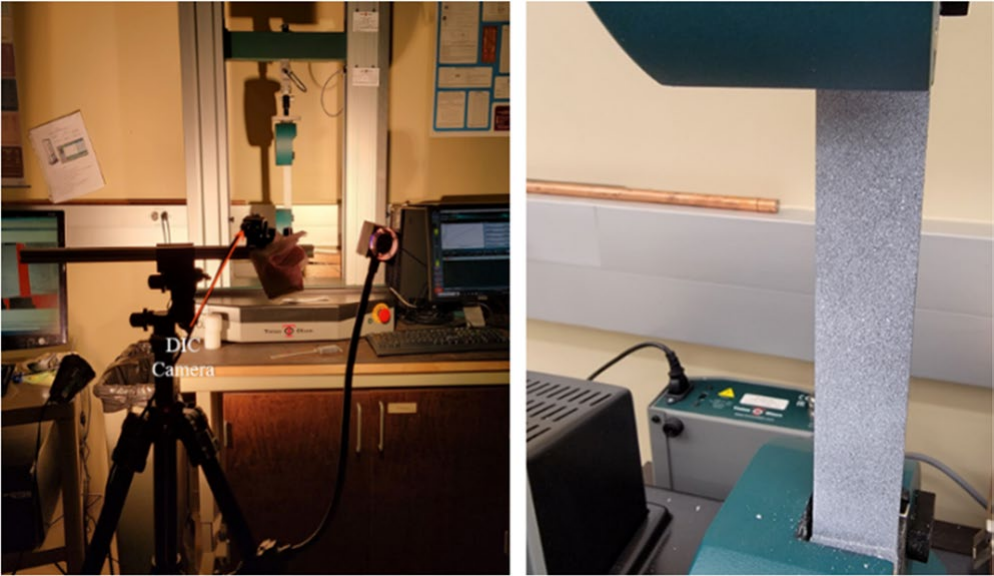
Experimental Setup for the ± 45° in-plane shear test (left) and sample with random speckle pattern (right)

A Tinius Olsen testing machine was used to test the samples at a constant crosshead displacement rate of 0.02 mm/sec (Fig. 7). A digital image correlation (DIC) system from Correlated Solutions was employed to measure shear strain across the surface of the samples. To implement DIC, a speckle pattern was applied to the surface of the test samples, which was used as a reference surface for measurement. A camera lens (1001960 XNP 35–0901 from Schneider Kreuznach) captured high-resolution images at 500 milliseconds intervals per frame. Figure 7 shows the setup of in-plane share test with DIC system. The In-plane shear stress, according to ASTM D-3518 [36], the in-plane shear stress ($\:{\tau\:}_{xy}$) is half of tensile stress ($\:{\sigma\:}_{x}$) (i.e., since $\:{\sigma\:}_{y}=0)$, and the in-plane shear strain ($\:{\gamma\:}_{xy}$) is the subtraction of x directional tensile strain ($\:{\varepsilon\:}_{x}$) and y directional tensile strain ($\:{\varepsilon\:}_{y}$) (i.e., due to the Passion’s ratio $\:{\nu\:}_{xy}=-{\varepsilon\:}_{y}/{\varepsilon\:}_{x}$). Five specimens for each type of CFRP test case, having dimensions of 203.2 mm (8.0 inch) in length and 25.4 mm (1.0 inch) in width were tested in compliance with the ASTM standard to get the in-plane shear strength.        

1 $${\tau\:}_{xy}=\frac{{\sigma\:}_{x}}{2}$$

2 $${\gamma\:}_{xy}=\:{\varepsilon\:}_{x}\:-\:{\varepsilon\:}_{y}$$

Using the Eq. (1), the in-plane shear stress was calculated from tensile stress. Using the Eq. (2), the shear strain was calculated from the x-directional and y-directional tensile strains taken from the DIC data.

## Results and discussion

### In-plane shear strength and modulus

**Fig. 8:**
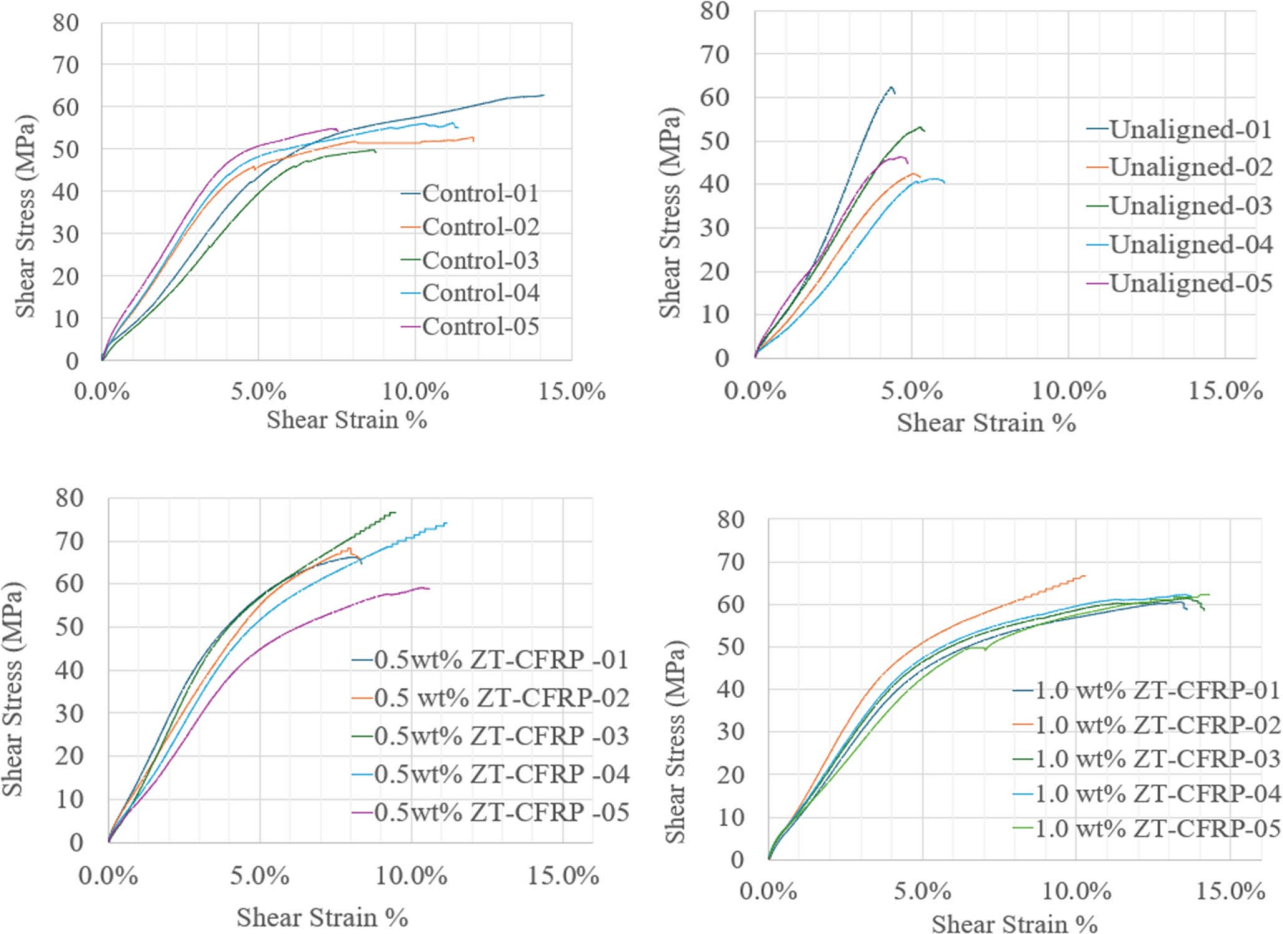
In-plane shear stress vs. strain curves of control CFRP (top left), unaligned 0.5wt% CNF-modified CFRP (top right) 0.5 wt% ZT-CFRP (bottom left) 1.0 wt% ZT-CFRP (bottom right)

Figure 8 presents the in-plane shear stress vs. strain curves for all types of laminate samples. Five specimens from each type of test case were tested according to ASTM standard and the stress vs. strain curves are plotted. Using the control CFRP samples’ curves as the baseline, the unaligned CNF-modified CFRP showed weaker and significantly more brittle failure, where the 0.5 wt% ZT-CFRP and 1.0 wt% ZT-CFRP samples showed significantly improved in-plane shear strength and toughness along with improved performance consistency. This reinforces the previous statement that the multiscale interlocked networks of zigzag z-threaded CNFs improve the nano-enhanced bonding (or z-threading locking) among the layers of ZT-CFRP and give higher bonding strength/energy to sustain while load is applied. To get more detailed value comparison, Table 1 describes statistics of the in-plane shear properties of control CFRP, 0.5wt% unaligned CNF-modified CFRP, and 0.5wt.% and 1.0 wt% ZT-CFRPs at the as-produced carbon fiber volume fractions and the normalized fiber volume fractions (assumed to be 60 vol%) and the average standard deviation (SD) of in-plane shear strength and shear modulus are presented.

**Table 1:** Statistics of in-plane shear properties of control CFRP, unaligned CNF-modified CFRP, and ZT-CFRP laminates

CFRP Type:	Control CFRP	Unaligned 0.5wt% CNF-modified CFRP	0.5 wt% ZT-CFRP	1.0 wt% ZT-CFRP
Fiber Volume Fraction (%)	53	60	54	50
In-plane Shear Strength (MPa)	55.06	49.07	69.14	62.22
SD	4.91	4.21	2.25	2.44
Rel. Improvement	N.A.	−10.88%	25.57%	13.00%
In-plane Shear Modulus (GPa)	1.02	1.25	1.21	1.01
SD	0.13	0.42	0.21	0.14
Rel. Improvement	N.A.	22.55%	18.63%	−0.98%
In-plane Shear Strength at Normalized (60% Fiber) Volume Fraction (MPa)*	62.33	49.07	76.82	74.66
Rel. Improvement*	N.A.	−21.27%	23.25%	19.78%
In-plane Shear Modulus at Normalized (60% Fiber) Volume Fraction (GPa)*	1.15	1.25	1.34	1.21
Rel. Improvement*	N.A.	8.25%	16.43%	4.96%

*Rough estimation based on fiber-dominating assumptions and may include inaccuracy

From Table 1, the average in-plane shear strength for the 0.5wt.% ZT-CFRP, 1.0 wt% ZT-CFRP, control CFRP, and unaligned 0.5wt% CNF-modified CFRP are 69.14 MPa, 62.22 MPa, 55.06 MPa, and 49.07 MPa, respectively. This means that both 0.5wt% and 1.0wt% ZT-CFRP had higher in-plane shear strengths than regular CFRP, and the unaligned CNF-modified CFRP had the lowest in-plane shear strength. The better performance indicates that the CNF z-threads reinforce the layers by forming an interlocked multiscale fiber reinforcement network. The 0.5wt% ZT-CFRP had higher in-plane shear strength than the 1.0 wt% ZT-CFRP likely due to the less interference between the diluted CNFs and promote a better alignment control of CNF z-threads during the prepreg manufacturing process. By comparing the in-plane shear strengths of unaligned 0.5wt% CNF-modified CFRP, 0.5wt% and 1.0wt% ZT-CFRP, one can conclude the alignment control of CNFs played an important role in the reinforcing system. The 0.5wt% ZT-CFRP provided the highest in-plane shear strength with a significant 25.57% increase from the control CFRP. The unaligned CNF-modified CFRP caused the lowest in-plane shear strength with a 10.34% reduction from the control CFRP. If for 60% carbon fiber volume fraction normalized in-plane shear strength, the changes by the 0.5wt% ZT-CFRP and the unaligned 0.5wt% CNF-modified CFRP against the control CFRP are estimated to be about + 23.24% and − 21.27%, respectively. It is necessary to note that these normalized estimations may not be accurate since they are based on the assumption that the carbon fibers are the major load bearing component responsible for carrying the mechanical load for the in-plane load on the CFRP laminate, and the in-plane shear strength and modulus of the laminate are assumed to be directly proportional to the carbon fiber volume fraction; this rough normalized estimation will not be able to count the matrix stress-concentration and the matrix/fiber interaction nor crack propagation/arrest in the laminates, and could only be treated as rough projections rather than the validated experimental data.

The average in-plane shear moduli of 0.5wt.% ZT-CFRP, 1.0 wt% ZT-CFRP, control CFRP and unaligned 0.5wt% CNF-modified CFRP are 1.21 GPa, 1.01 GPa, 1.02 GPa, and 1.25 GPa, respectively (see Table 1). The in-plane shear modulus changes of the 0.5wt% ZT-CFRP (the highest) and the 1.0wt% ZT-CFRP (the lowest) compared with the control CFRP are + 18.63% and − 0.98%, respectively. Comparing all types of samples, adding CNFs in the CFRP usually increased, or at least did not notably reduce, the in-plane shear modulus.

**Fig. 9:**
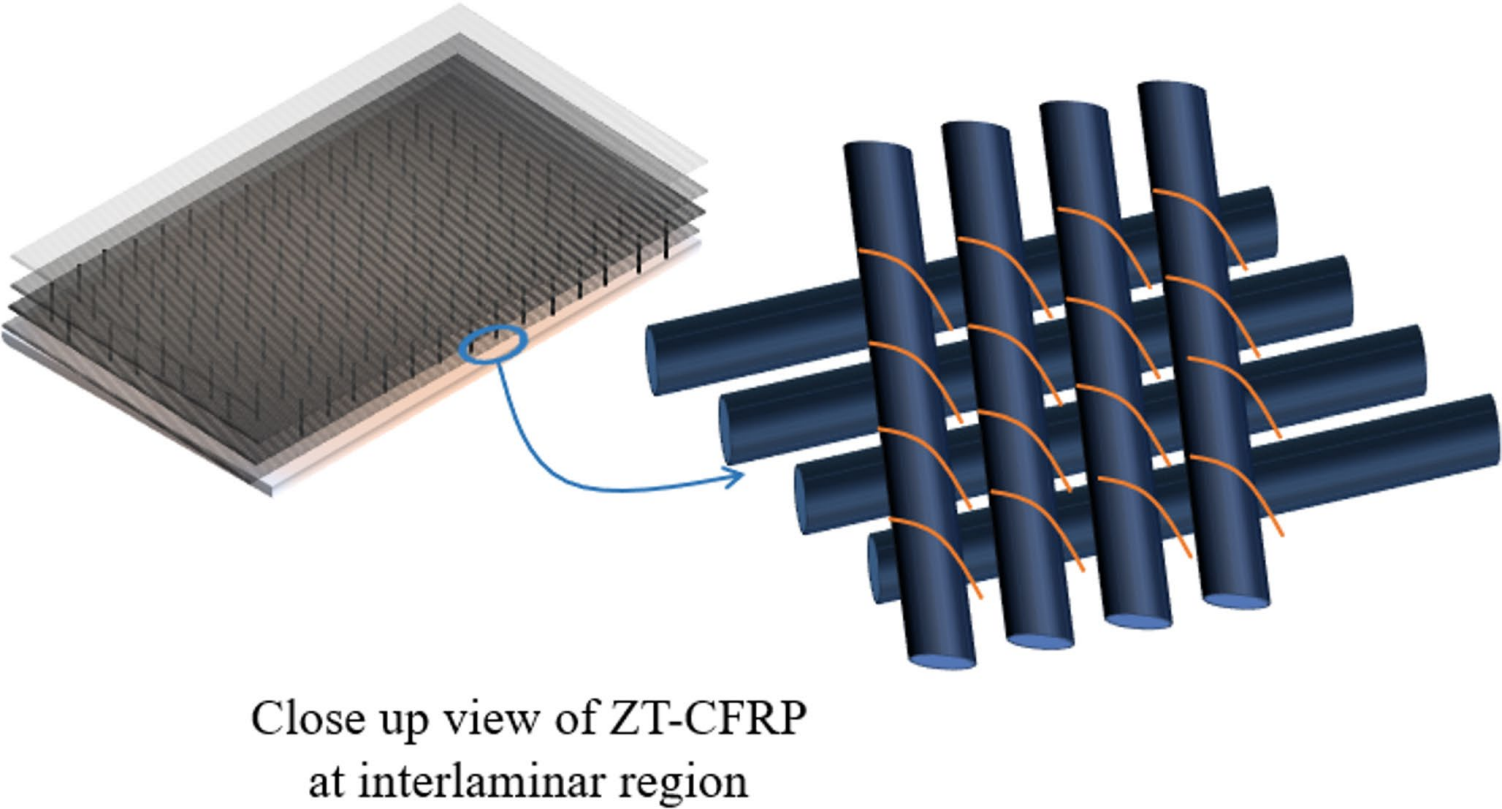
Illustration of aligned CNF z-threads at the interlaminar region of ZT-CFRP

In Fig. 8, ZT-CFRP specimens showed more consistent shear stress in their stress-strain curves than the control and unaligned CNF-modified CFRP specimens. The properties of CFRP in elastic region depend primarily on the fiber volume fraction. In the plastic region, the carbon fibers start to be scissoring, and the nanofibers present in the ZT-CFRP specimens (also see Figs. 1 and 5) resist the scissoring and ultimately provide better shear resistance than the control CFRP specimens that have no nanofiber reinforcement. In the stress-strain curve, there is non-linearity at the beginning of the test which might be due to the tightening of the wedge-type clamp or slight sliding of the samples at the beginning of the testing process. The control and unaligned samples transitioned into the plastic region differently than the ZT-CFRP samples. This variation is because of the initiation of carbon fibers scissoring/rotation and delamination in the interlaminar region or/and the intralaminar region. In the elastic region, the mechanical properties of CFRP are primarily determined by the fiber volume fraction and the properties of the resin matrix. As a result, the in-plane shear modulus of both control CFRP and ZT-CFRP (CFRP reinforced with aligned Z-threaded Carbon Nanofibers, CNFs) are almost identical under these conditions. However, the real advantages of ZT-CFRP come into play once the material enters the plastic deformation region. At the plastic deformation stage, the carbon fibers in the ± 45° orientation of the CFRP begin to experience significant scissoring/rotation under shear stress. This carbon fiber shear rotation is a key factor in the material’s failure; and the crack can be initiated at any weak point under high shear stress whether at the interlaminar region or intralaminar region. However, the unique structure of aligned Z-threaded CNFs plays a pivotal role in resisting this carbon fiber rotation. The CNFs, with their specific alignment and zigzag thread-like form, act as reinforcing bridges within the matrix, providing significant resistance to the rotation of the fibers and help to distribute the load to the CNF z-threaded carbon fibers. This resistance effectively enhances the material’s in-plane shear strength, allowing ZT-CFRPs to maintain structural integrity and resist further deformation. Figure 9 illustrates the critical role of aligned Z-threaded CNFs in enhancing the in-plane shear strength in CFRP at the interlaminar region of a [± 45°] ZT-CFRP laminate. The presence of these nanofiber z-threads offers more than just mechanical reinforcement; they form a network of bridges that significantly restrict the rotational movement of the + 45° and − 45° microfibers, thereby contributing to the material’s superior shear resistance in the plastic range. Based on the experimental data of Table 1; Fig. 8 along with the illustration of Fig. 9, one can understand the alignment and properties of the CNFs along with the z-threaded nano-microstructural patten help to create stronger, tougher, more effective, and nano-enhanced bonds/reinforcement when the laminate is under in-plane shear loading. However, more detailed discussion about the micro-level stresses and failure mechanisms in the interlaminar region and intralaminar region will also shed further insight for the future development of advanced nanostructured CFRP laminated structures.

For a [± 45°]_ns_ traditional CFRP laminate, the in-plane shear strength is characterized as the maximum $\:{\tau\:}_{xy}$ carried by the overall laminate during the ASTM D-3518 test, and the first failure site can occur at interlaminar region or intralaminar region within the laminate. Based on the lamination theory, the lamina strains of all plies are identical to the laminate strains for this given ply-sequence and loading condition. However, within and near the interlaminar region or interface (if one assumes the region has negligible thickness but may not be a precise assumption), the significant mismatch of the elastic properties between the + 45° lamina, the − 45° lamina, and the resin matrix (i.e., the thin resin layer between the two laminas) will cause a lot of stress rising and can be a first failure site initiated with the apparently weaker points from the microscopic view such as the resin matrix and/or the resin-fiber interface. Furthermore, near the edge of the [± 45°]_ns_ sample, due to the laminate’s edge effect, which is known to cause the laminate’s interlaminar stresses ($\:{\sigma\:}_{zz}$, $\:{\tau\:}_{xz}$, and $\:{\tau\:}_{yz}$) rise abruptly near the edge’s interlaminar region and may initiate interlaminar failure from the edge. As shown in Fig. 9, within and near the interlaminar region a [± 45°]_ns_ ZT-CFRP laminate, the long CNF z-threads will act as effective nano-reinforcements by mechanically bridging the + 45° lamina and the − 45° lamina, and will help reduce the stresses acting on the resin and resin-fiber interface or serve as microcrack arresters. Therefore, the CNF z-threads can contribute to an effective micromechanical role against the interlaminar failure during the in-plane shear test. However, it is important to note that the length, diameter, alignment status/angle, mechanical properties and concentration of CNFs, as well as the polymer matrix’s mechanical and adhesion/wetting properties will also affect the performance against interlaminar region’s shear failure.

Different from the interlaminar failure consideration, the intralaminar failure mechanics will require shifting the analysis focus to the individual laminas of the [± 45°]_ns_ laminate. Each lamina, i.e., an intralaminar region, consists of unidirectional carbon fibers with the lamina’s longitudinal direction (i.e., 1-direciton) all aligned in either the + 45° or −45° against the global x-direction of the laminate; these parallelly packed carbon fibers are connected by the matrix and matrix-fiber interface. When a [± 45°]_ns_ traditional CFRP laminate is subjected to the in-plane shear test, the parallelly packed carbon fibers in a lamina are carrying a high longitudinal tensile load in the 1-direciton (due to the high elastic modulus in the 1-direciton), a low transversal load in the 2-direction (due to the low elastic modulus in the 2-direciton), and a 1–2 directional shear load. From the lamination theory, the directions of the longitudinal tensile stresses and the 1–2 directional shear stresses of the + 45° lamina and the − 45° lamina are different (due to their fiber alignment angle difference and the lamina’s stiffness matrix difference), hence will cause a significant stress gradient along the z-direction (i.e., 3-direction). On the other hand, due to the edge surface of a lamina is in contact with the air, such a boundary will cause a significant shear stress gradient in the y-direction as well as the 2-direction. Given the stress balance requires $\bf \:\nabla\:\bullet\:\boldsymbol{\sigma\:}=0$ in a lamina’s 1–2−3 coordinates system, from the point of view of the order of magnitude (OM) analysis, it will yield $\:OM(\partial\:{\tau\:}_{13}/\partial\:{x}_{3})\sim OM(\partial\:{\tau\:}_{12}/\partial\:{x}_{2})\ne\:0$. Therefore, in a lamina, near the edge of the laminate (or edge of any significant internal defects such as void or large impurity inclusion to cause significant gradient of $\:\partial\:{\tau\:}_{12}/\partial\:{x}_{2}$) there will be significant 1–3 (i.e., 1-z) directional shear stress $\:{\tau\:}_{13}$. Furthermore, it is hypothesized that the $\:{\tau\:}_{13}$ in a lamina is highest at the z-location immediately adjacent to the interlaminar region and gradually reduces to zero at the center-plane of the lamina (i.e., the symmetric plane requires $\:\partial\:{\tau\:}_{13}/\partial\:{x}_{3}=0$ and will be the location to have the minimum of $\:{\tau\:}_{13}$). The 1–3 directional shear strength of a unidirectional CFRP laminate can be characterized by the interlaminar shear strength (ILSS) of the laminate under a short beam shear tests. A previous study [37] reported that the ILSS of a unidirectional CFRP laminate can be significantly improved by the addition of CNF z-threads. Table 2 shows that control CFRP and unaligned CNF-modified CFRP (0.5 wt% and 1.0 wt%) had significantly lower ILSS than the corresponding ZT-CFRP (0.5 wt% and 1.0 wt%). Unidirectional ZT-CFRP laminates of 0.5wt% and 1.0wt% CNF concentration showed 8.20% and 17.00% improvement in ILSS compared to control CFRP, respectively, while unaligned CNF-modified CFRPs showed minimal improvement. Therefore, from the micromechanical analysis view, the assumed pure intralaminar failure (assuming if the interlaminar region were controlled and not allowed to fail first) of an in-plane shear failed [± 45°]_ns_ CFRP or ZT-CFRP laminate could be somewhat related to the ILSS of a unidirectional CFRP or ZT-CFRP laminate, respectively.

**Table 2:** Statistics of interlaminar shear properties of control CFRP, unaligned CNF-modified CFRP, and ZT-CFRP laminates [37]

CFRPs	Control CFRP	Unaligned 0.5 wt% CNF-modified CFRP	Unaligned 1.0 wt% CNF-modified CFRP	0.5 wt% ZT-CFRP	1.0 wt% ZT-CFRP
Fiber Volume Fraction (%)	60	61	59	60	61
Interlaminar Shear Strength (ILSS) (MPa)	64.89	65.10	67.74	70.21	75.92
SD	2.2	2.2	2.73	1.61	4.96
Rel. Improvement of ILSS	N/A	0.32%	4.39%	8.20%	17.00%

Based on the discussion of interlaminar and intralaminar failure mechanisms, if one further compares the values of in-plane shear strength of [± 45°] CFRP laminate (Table 1) and the ILSS of unidirectional CFRP (Table 2), he/she can find their magnitudes are in the similar range (about 50–80 MPa). Compared with traditional CFRP laminate, one can also find ZT-CFRP have significant improvements in the in-plane shear strength of [± 45°] laminates and the ILSS of unidirectional laminates, while unaligned CNF-modified CFRP have negative or negligible improvements. However, the unidirectional laminates’ ILSS improvement cannot be fully translated to the [± 45°] laminates’ in-plane shear improvement due to some other terms not considered in this simplified analysis (e.g., resin-fiber interface debonding, Poisson’s ratios effects, transversal stress, CNF’s effectiveness to alter the stress and strain distribution in the resin under different mechanical loading directions). In addition, some non-perfectly aligned long CNFs in a ZT-CFRP lamina may be tilted along the carbon fiber direction thus reduce the effectiveness of mechanical stress-transfer with the adjacent perpendicularly aligned ZT-CFRP lamina as shown in Fig. 9 when taking an in-plane shear stress. On the other hand, such non-perfectly aligned long CNFs (i.e., partially tilted along carbon fiber direction) in a unidirectional ZT-CFRP lamina can still effectively carry the 1-z directional shear stress and improve the ILSS. In some scenarios, one could even argue weather a perfectly z-aligned long CNF z-threads or a slightly tilted (e.g., 45° tilted) CNF z-threads can take 1-z directional shear load more effectively if assuming the CNFs are not buckled and are well supported by the resin. The in-plane shear strength of a [± 45°] ZT-CFRP laminate will depend on both the strength at the interlaminar region, the strength at intralaminar region, the stress gradients or distribution along the z-direction, and the micro-level stress distribution among the resin, CNFs, interface, and carbon fibers. This area could be a future new research direction in terms of experimental approaches (characterization and manufacturing process advancement) and modeling methods (numerically or/and analytically) and will play a critical role to open a new page to utilize the advantages of next-generation advanced nanostructured composites such as the ZT-CFRP.

### Strain energy density analysis of laminate samples

The in-plane shear strength and shear modulus are higher at 0.5 wt% ZT-CFRP. It was also observed in a previous study that lower wt% concentration of CNF z-threads had better performance than higher concentration of CNFs [23] as the higher CNFs concentration possibly increased the chance of CNF local interference/percolation while plural of CNFs being aligned and threaded through the space between carbon fibers during the manufacturing steps. However, the roles of CNFs in a ZT-CFRP laminate may not be only limited to those well-aligned Z-threads. Partially z-aligned or not z-aligned CNFs in the ZT-CFRP system may also affect the fracture process, especially some fracture mechanisms and fracture energy involving CNF fracture or/and pullout. Therefore, it would be interesting to calculate the strain energy density (SED) for the tested samples to understand the contribution of CNFs to the fracture energy. Equations 3–5 are used for the strain energy density calculation.

3 $$\:\mathrm{I}={\int\:}_{0}^{\varepsilon}\mathrm{d}\mathrm{I}$$

4 $$\:\mathrm{d}\mathrm{I}=\sigma\:\mathrm{d}\varepsilon$$

5 $$dI\cong\left(\frac{\sigma_i+\sigma_{i+1}}2\right)\left(\varepsilon_{i+1}-\varepsilon_i\right)$$

 

where I, σ and ε are strain energy density, stress, and strain, respectively.

Table 3 describes the strength energy density of the tested samples. The modulus of toughness (i.e., the total strain energy covering elastic range and plastic range) of control CFRP, unaligned CFRP, 0.5 wt% ZT-CFRP and 1.0 wt% ZT-CFRP are 4.40 MJ/m^2^, 1.64 MJ/m^2^, 4.60 MJ/m^2^, 5.38 MJ/m^2^, respectively. Compared with the modlus of toughness of the control CFRP, the changes of unaligned 0.5wt% CNF-modified CFRP, 0.5wt% ZT-CFRP, and 1.0wt% ZT-CFRP are − 62.73%, + 4.54%, + 22.27%, respectively. The 1.0wt% ZT-CFRP performed the best while the unlainged CNF-modified CFRP became much more brittle compared with the control CFRP, as one can aslo expect from the stress-strain cruves shown in Fig. 8.

**Table 3:** Average Strain Energy Density of control CFRP, unaligned CFRP and ZT-CFRP laminates

CFRP Type:	Control CFRP	1.0 wt% Unaligned CFRP	0.5 wt% ZT-CFRP	1.0 wt% ZT-CFRP
Modulus of Resilience (MJ/m^2^) = I_total_ (Elastic range)	1.34	0.92	1.06	0.83
Strain Energy Density at Plastic Region (MJ/m^2^)	3.07	0.72	3.53	4.55
Modulus of Toughness (MJ/m^2^) = I_total_ (Elastic range) + I_total_ (Plastic Range)	4.40	1.64	4.60	5.38
Standard Deviation	1.31	0.12	0.84	0.54
Rel. Improvement of Modulus of Toughness	N.A.	−62.73%	4.55%	22.27%

The modulus of resilence, (i.e., strain energy density in the elastic range) of control CFRP, unaligned 0.5wt% CNF-modified CFRP, 0.5 wt% ZT-CFRP and 1.0 wt% ZT-CFRP are 1.34 MJ/m^2^, 0.92 MJ/m^2^, 1.06 MJ/m^2^, and 0.83 MJ/m^2^, respectively. The control CFRP performed the best in the modulus of resilence to facilitate the elastic energy storage in its fiber-resin system. In the plastic region, the SED of control CFRP, unaligned CFRP, 0.5 wt% ZT-CFRP and 1.0 wt% ZT-CFRP are 3.07 MJ/m^2^, 0.72 MJ/m^2^, 3.53 MJ/m^2^, 4.55 MJ/m^2^, respectively. Unaligned CFRPs showed the lowest plastic range SED while 1.0 wt% ZT-CFRP showed the highest, which was also exhibited in the stress-strain curves in Fig. 8. Although shear strength is higher in the case of 0.5 wt% ZT-CFRP, its SED performance is lower than that of 1.0wt% ZT-CFRP. This suggests that while the CNF z-threading reinforcement enhance shear load-bearing capacity, the lower CNF content at 0.5wt% and possibly sub-optimal alignment result in less overall deformation and energy absorption capacity than the 1.0wt% ZT-CFRP case. The 0.5wt% ZT-CFRP becomes relatively stronger but less tough than the 1.0wt% ZT-CFRP, leading to a lower strain energy density in the plastic region, and ultimately lower the moduls of toughness. On the other hand, the unaligned 0.5wt% CNF-modified CFRPs, regardless of filler content, show the lowest SED due to the lack of reinforcement orientation and many unaligned CNFs may cause stress concentration during the fracture of the laminate samples.The data provide interesting insights about the distinct importance of the alignement of CNFs in the CFRP laminates, espcailly the Z-threaded composite architecture. Further future experiments or numerical simulations on this topic can give a better understanding of these behaviors.

**Fig. 10:**
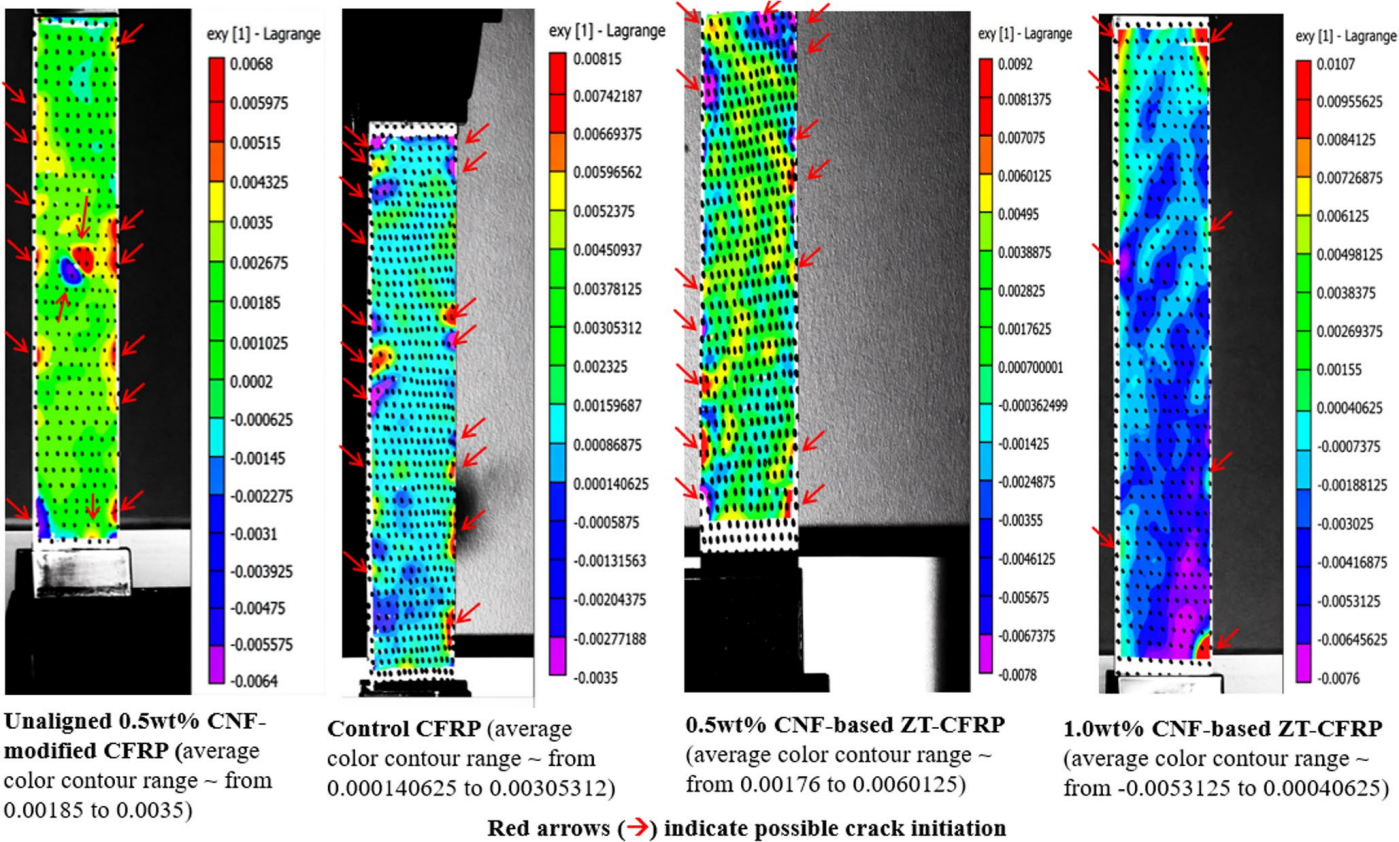
Comparison of the DIC strain distribution images during the in-plane shear test with similar range of average value of in-plane shear strain magnitude for unaligned 0.5wt% CNF-modified CFRP, control CFRP sample, 0.5 wt% CNF-based ZT-CFRP and 1.0 wt% CNF-based ZT-CFRP sample (from left to right); The areas with the strain contour value significantly deviated from the average value and with well-defined boundary suddenly jump from the surrounding (like a pocket) are marked as possible delamination areas. Possible delamination areas for the unaligned CNF-modified CFRP were the center crack and edge crack propagation. For control CFRP, the possible edge crack propagation was found. ZT-CFRP’s had possible edge crack but not showing any significant crack propagation except near the clamp/tab ends

The nano-reinforcement provided by the CNF-z threads reduces the likelihood of delamination propagation or slipping under shear stress which are common failure modes in composite materials. The aligned CNF Z-threads provide a distinct reinforcement at the fiber-matrix interface, preventing the formation and propagation of micro-cracks and other premature failure that typically arise during shear deformation of a traditional CFRP and an unaligned CNF-modified CFRP. Figure 10 shows the shear strain distribution of the four types of laminates recorded at similar ranges of average shear strain magnitude (i.e., the color code of the majority of area). It was found that the highest and the lowest shear strains that most deviated from the average value tended to concentrate at weak points especially along the laminate’s edge (as known for lamination’s edge effect as well as possible damage or resin heating caused by sample cutting), leading to potential failures as crack propagation which are evident in the DIC strain distribution. Comparing all four types of laminates’ DIC images, if neglecting the stress-concentration area near the clamps, one can see that unaligned 0.5wt% CNF-modified had the edge crack propagation and center crack formation/propagation. The control CFRP showed edge crack propagation. The 0.5wt% CNF-based ZT-CFRP and 1.0wt% CNF-based ZT-CFRP had edge crack but not showing significant crack propagation yet. Combining the information from Fig. 10; Table 1, and Table 3, one can yield the conclusion and better understanding that the CNF z-threads helped to sustain the edge strain better and delayed the delamination failure thus providing a higher in-plane shear strength and a higher modulus of toughness.

**Fig. 11:**
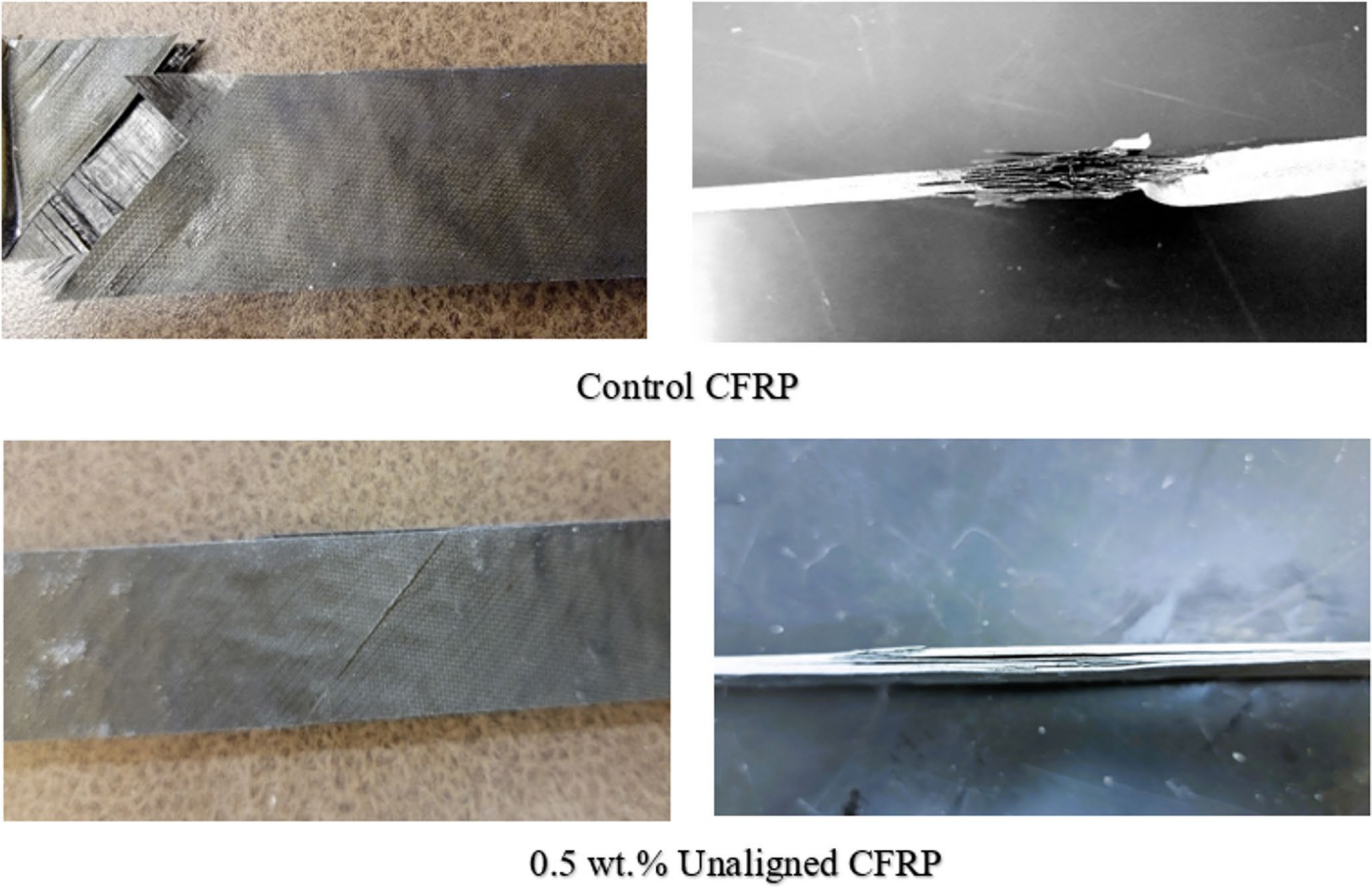
Failure mode after failure of control CFRP sample (top) and unaligned CNF-modified CFRP sample (bottom)

**Fig. 12:**
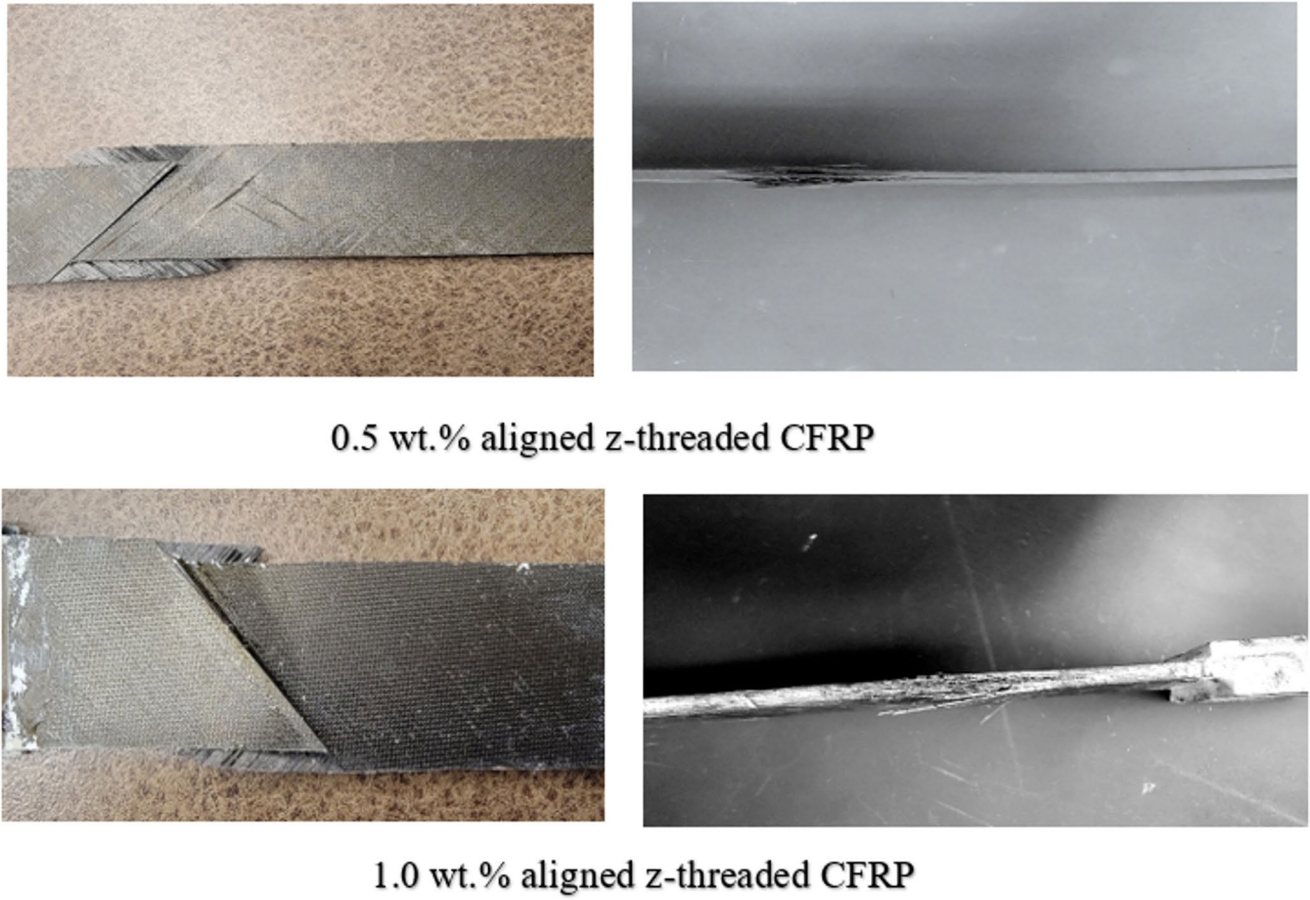
Failure mode after failure of 0.5 wt% ZT- CFRP sample (top) and 1.0 wt% ZT-CFRP sample (bottom)

From Figs. 11 and 12, one can visually compare the failure sites of all types of laminates. The unaligned CNF modified CFRP had very different failure mode than the others; the centerline-only delamination mode signified its brittle nature (as also shown in the stress-strain curve and the lowest modulus of toughness). The control CFRP, 0.5wt% ZT-CFRP, and 1.0wt% CFRP all had localized multiple-plies delamination through the thickness, however, the ZT-CFRP samples had substantial edge cracks jumping between the plies that indicated some enhanced bridging effects between the angle plies could be due to the CNF-z-threaded CFRP laminate structure (see Fig. 1). The failure mode observation can also support the enhanced in-plane shear strength and strain energy density (i.e., modulus of toughness) of ZT-CFRP.

The unique failure characteristics of ZT-CFRP, which exhibit significantly higher in-plane shear strength, further supports the argument that the interlaminar and intralaminar regions in ZT-CFRP are stronger than those in conventional CFRP. This enhancement is primarily attributed to the distinctive nano-structure caused by the CNF z-threads, which weave through the carbon fiber array. In traditional CFRPs, the adhesion between the laminas and the resin-fiber interface often represent weak points, which can lead to fiber-matrix separation/cracking and delamination within or/and between the layers under stress. In contrast, the zigzag threading of the CNFs in ZT-CFRPs (see Fig. 1) reinforce the connections between the carbon fibers as well as between the laminas, and mitigates the premature separation within and between the prepreg plies under in-plane shear load, thereby improving the material’s resistance to delamination and other failure modes as quantitively presented through the significantly enhanced in-plane shear strength and modulus of toughness. To further understand the roles of CNF z-threads, it would be helpful to compare its results with the other reported CNT-based in-plane shear strength related studies. Table 4 shows a summary of CNT-based studies conducted on the improvement of in-plane shear strength or other in-plane strength related properties, including this study and the works by others for an overall comparison and understanding. Note that De Villoria et al. [27]. didn’t report direct measurements of in-plane shear strength of [45/−45]_4 S_ laminate according to the ASTM D-3518 [36] like what was tested in this paper, but some of the reported in-plane strength related improvements of their 16-ply [(0/90/±45)_2_]_s_ quasi-isotropic laminate sandwiched with 15 plies of aligned CNF forest interfaces can imply the likelihood of in-plane shear strength improvement due to aligned CNTs. From Table 4, one can conclude that the use of unaligned nanomaterials has little impact on in-plane shear strength. On the contrary, aligned CNT (or CNF) approaches, including the aligned CNTs forest interface/interleaves apprach (i.e., the nano-stitching approach) [27] and this low concentration (0.5wt%) ZT-CFRP prepreg approach both showed substantial improvement in the in-plane shear related properties improvement. Furthermore, different than the aligned CNT forest interleave approach reported by De Villoria et al. [27] that needs a special processing step to be selectively placed between two plies of prepregs during a laminate manufacturing process, the low concentration CNF-based ZT-CFRP prepreg, produced by the roll-to-roll hot-melt production method, has the other distinct advantages of easy-to-use (i.e., the ZT-CFRP prepregs were handled exactly same as regular CFRP prepregs during the storage and laminated parts manufacturing), scalable, and low cost; the performance could be further enhanced if the CNFs could be replaced with long CNTs in the future.

**Table 4:** Summary of CNT/CNF-based studies conducted to improve in-plane shear properties of CFRP

References	Fiber Type	Method	Epoxy Resin	Result
[17]	CYCOM 5320 T40/800	Unaligned CNFs coating	Araldite MY 0510	No improvement of in-plane shear strength was observed but failure modes were different.
[18]	AS4	Unaligned CNTs coating	EPON 862	Prepreg specimens with CNT mats showed lower in-plane shear strength than baseline specimens.
[19]	Unidirectional glass fiber	Unaligned CNTs coating	L20	Addition of MWCNT did not improve the in-plane shear properties.
[20]	Carbon Woven Fabrics	Unaligned CNTs coating	Araldite LZ 5021	A slight improvement was observed.
[27]	AS4	Aligned CNTs Forest interfaces/interleaves placed between prepregs	Hexcel 8552	Bolt bearing strength (i.e., tension bearing strength), open-hole compression strength, L-shape bending strength improved by 30%, 14%, and 26%, respectively.
This study	AS4	Low concentration (0.5wt%) CNF based ZT-CFRP prepreg	EPON 862	25.57% increase in the in-plane shear strength.

### Microscopic analysis

**Fig. 13:**
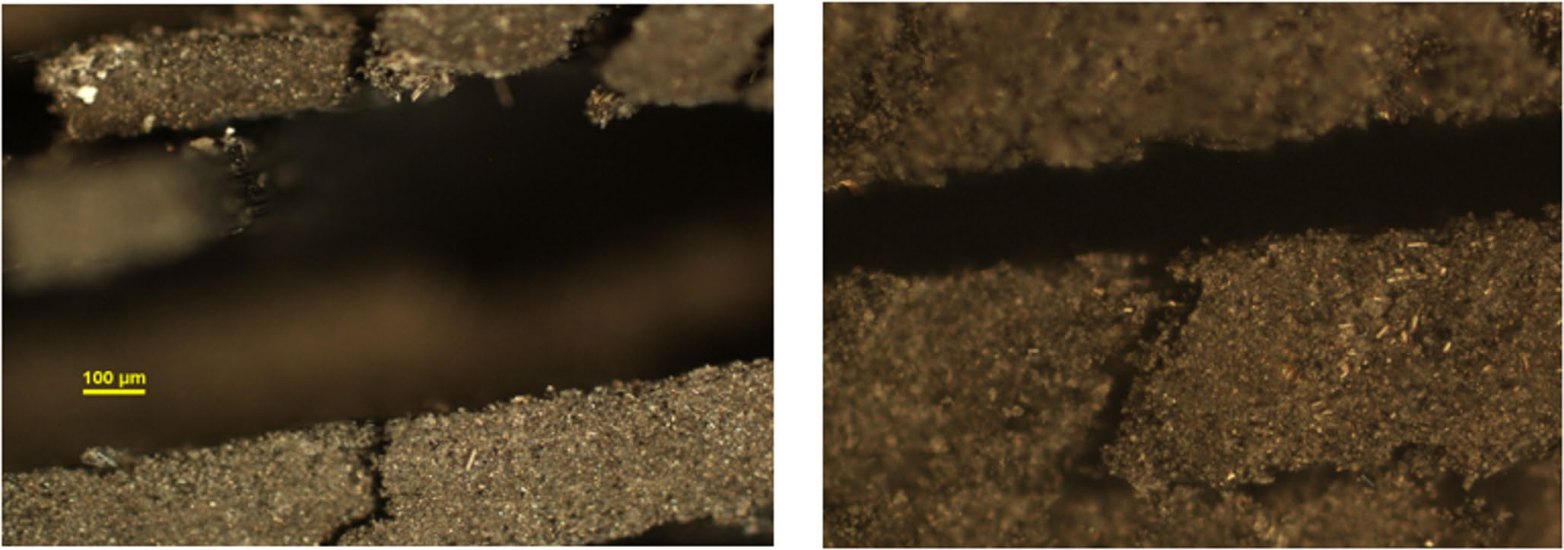
Microscope images of fractures surfaces for unaligned CNF-modified CFRP samples along the side profile at 100x (left) and 200x (right) magnification

**Fig. 14:**
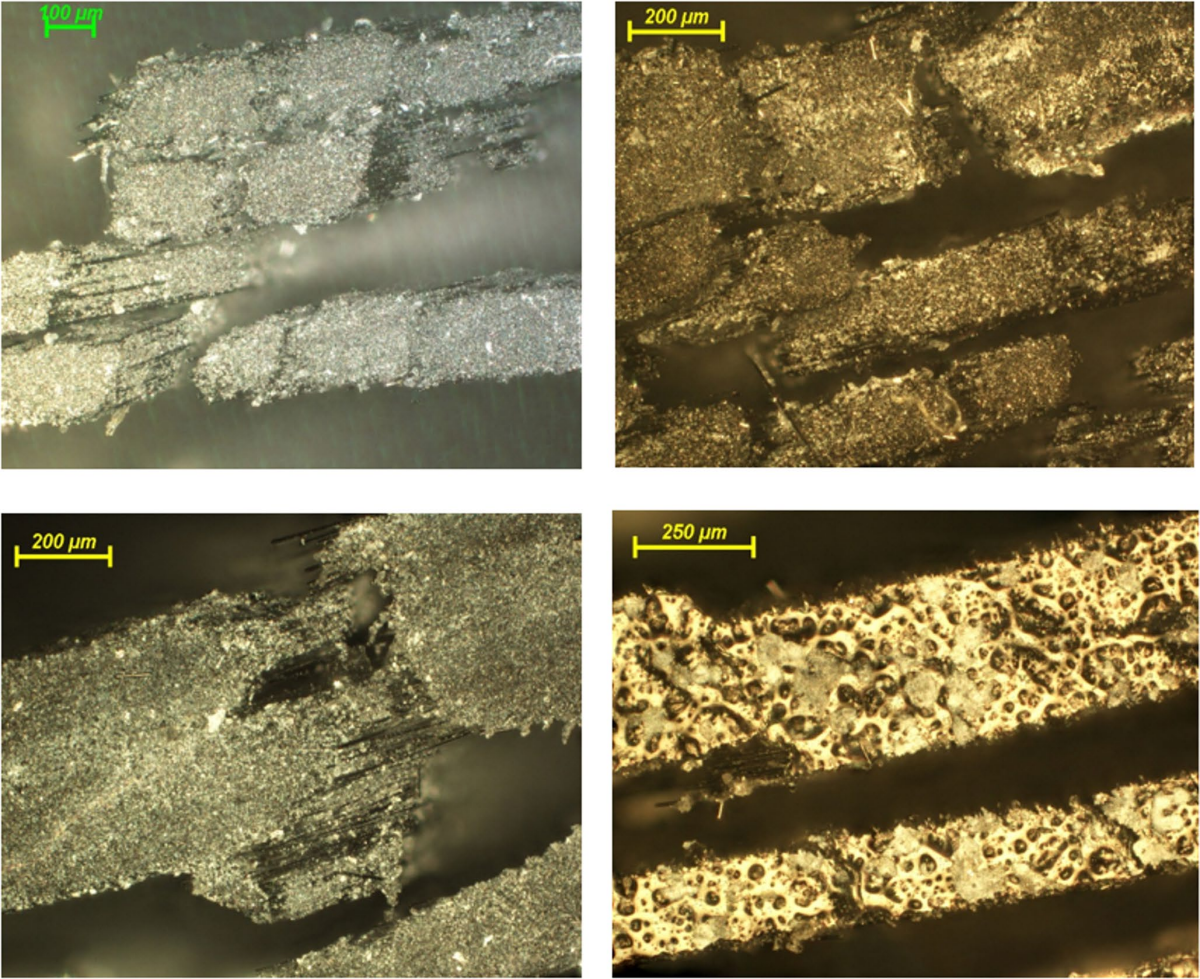
Microscope images of fractured surfaces for control CFRP (top) and ZT-CFRPs (bottom) samples along the side profile at 100x magnification

**Fig. 15:**
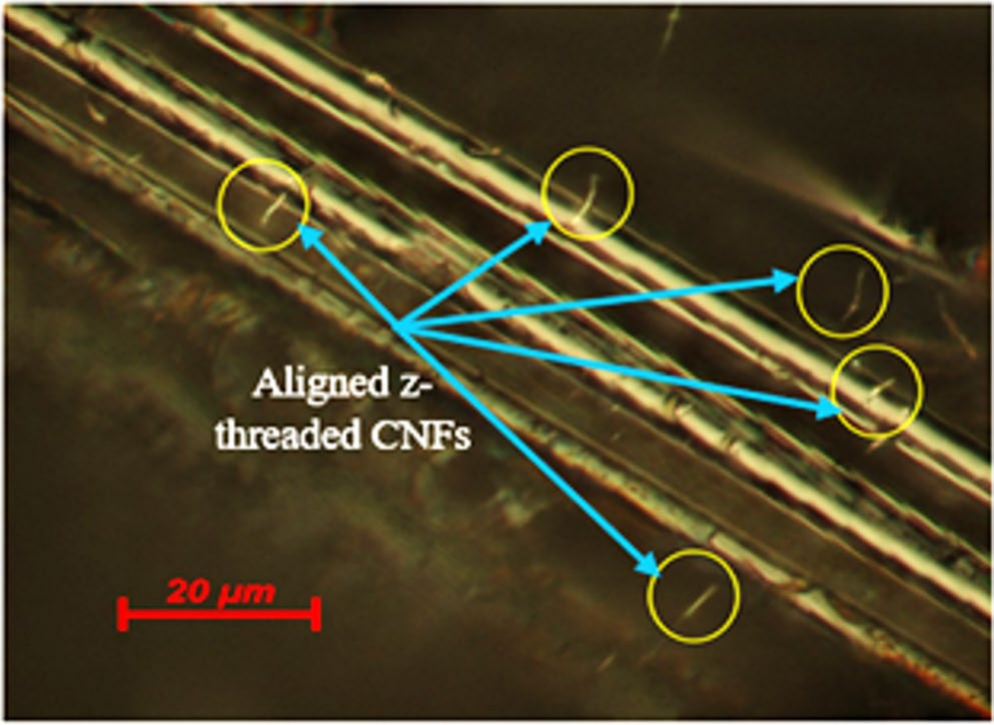
Microscope images of fractured surface for a ZT-CFRP sample along the side profile and presence of zigzag z-threaded nanofibers at 1000x magnification

The specimens tested were examined under a microscope (Nikon Eclipse LV 150) for a clearer view of the delamination failure and identify any potential differences. The unaligned CNF-modified CFRP sample (see Fig. 13) showed simple and straight crack propagation, which indicated its brittle characteristic against the in-plane shear delamination. Figure 14 shows the fracture sites’ side profile view for control CFRP and ZT-CFRP specimens. Both control CFRP and ZT-CFRP had complex delamination fractures, which could be interlaminar delamination or intralaminar cracking. If one compares them in more detail, one can observe that the control CFRP had many pull-away or missing pieces along the same layer(s), which indicated more intralaminar cracking fracture sites. On the other hand, the ZT-CFRP had significantly less amount of missing pieces along the same layer(s) suggested that it had a stronger resistance against and suppressed the intralaminar fracture. Combining the microscopy comparison with the significantly improved in-plane shear strength of ZT-CFRP (see Table 1; Fig. 8), one can conclude that the CNF z-threads in each prepreg helped resist the intralaminar fracture by acting like anchors between the carbon fibers. The control samples show extensive fiber pull-out and discontinuous fracture surfaces in a layer, which indicate the characteristics of intralaminar fracture. In contrast, ZT-CFRP samples exhibit more cohesive fracture surfaces with significantly reduced fiber pull-out, fewer fracture segments which indicating less intralaminar fracture. Figure 15 shows the 1000 times magnified microscope picture of the side profile of a fractured ZT-CFRP sample; one can see the fractured and slightly pulled out CNFs z-threaded among the carbon fibers of a ZT-CFRP ply. One can also see the pull-out length of CNFs are almost shorter than the diameter of carbon fiber (about 7 microns for AS7 carbon fiber), which was due to zigzag threading pattern and mechanical interlock between the long CNFs and the carbon fiber array.

**Fig. 16:**
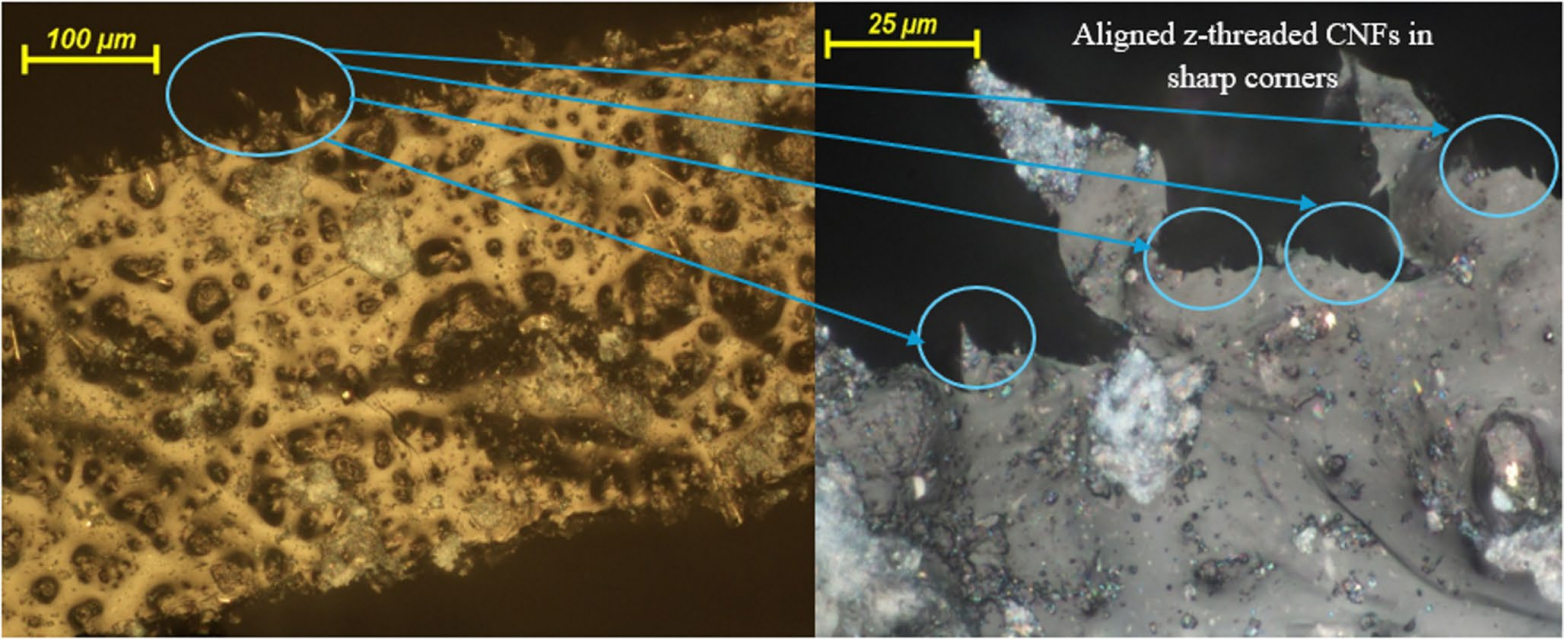
Microscope images of fractures surfaces for ZT-CFRP samples along the side profile and presence of zigzag z-threaded nanofibers at 200x (left) and 1000x (right) magnification

Microscopic images in Fig. 16 show many sharp spikes on the ZT-CFRP’s in-plane shear fracture surface; this means it is not easy to fracture as plain surface like control CFRP, and more force is required to fracture the ZT-CFRP. The presence of some CNFs was also observed along or embedded within these sharp spikes, indicating that the CNFs reinforced the bonding between the plies, thereby contributing to higher in-plane shear strength and strain energy density when in-plane shear load was applied. The CNF z-threads and the shape spikes shown in Figs. 15 and 16 further support the previous claims, strengthening the argument that CNF z-threads enhance the material’s performance by improving the mechanical load transfer at both interlaminar and intralaminar levels and improve the resistance to in-plane-shear failure.

The microscopy analysis helps the understanding that several complex reinforcing and toughening mechanisms can occur at the interlaminar and intralaminar regions when an advanced nanostructured composite laminate such as ZT-CFRP is taking in-plane shear load. In the future, more understanding about how to optimally exploit ZT-CFRP’s complex reinforcing and toughening mechanisms will open doors for expanding the material sciences and engineering knowledge towards next generation multi-scaled composites manufacturing and design.

## Conclusion

The carbon nanofiber Z-Threaded CFRP (ZT-CFRP), which utilizing long CNFs to thread among carbon fibers to create a zigzag mechanically interlocked multiscaled fiber reinforcement architecture, has been proven to be able to significantly improve the in-plane shear properties of [45/−45]_4s_ composite laminates when a low concentration of CNFs being used (i.e., 0.5wt% to 1.0wt% CNFs dispersed in the epoxy matrix). To evaluate the effect of CNF Z-threads on the in-plane shear strength and the modulus of toughness (i.e., total strain energy density) of the CFRP laminates, four types of samples (including control CFRP, unaligned 0.5wt% CNF-modified CFRP, 0.5 wt% CNF-based ZT-CFRP and 1.0 wt% CNF-based ZT-CFRP) were fabricated and tested. The unaligned 0.5wt% CNF-modified CFRP specimens showed weakest and most brittle fracture behavior. Both 0.5wt% and 1.0wt% CNF-based ZT-CFRP were stronger and tougher than the control CFRP. The 0.5 wt% ZT-CFRP showed higher in-plane shear strength and in-plane shear modulus than the 1.0 wt% ZT-CFRP. This phenomenon was likely due to the 1.0 wt% CNF concentration reduced the dispersion and alignment quality of the CNF, thus reducing the effectiveness of CNF z-threads in the ZT-CFRP prepreg. Although shear strength and shear modulus are higher in the case of 0.5 wt% ZT-CFRP, the modulus of toughness (i.e., total strain energy) performance is lower than that of 1.0wt% ZT-CFRP. This suggests that while the CNF z-threading approach enhanced shear load-bearing capacity, the lower CNF content at 0.5wt% (and possibly sub-optimal alignment) resulted in the less overall maximum strain and modulus of toughness.

The microscope pictures of the fracture sites were analyzed to obtain more insights. The unalgined 0.5wt% CNF-modified CFRP had clean and straight centeline-delamiation, which explained why it was weaker and more brittile compared to the other types of samples. ZT-CFRP lamiante had much less intralmainar fracture sites compared with the control CFRP; along with the other microscope picture showed the CNF z-threaded among carbon fibers of a ZT-CFRP ply, one can see that the CNF z-threads significantly improved the resistanct against intralamianr delamiation. Along the delamination surface some shape spikes were also found and some CNFs were found in the spikes; these sharp spikes can explan why high in-plane shear stress and strain energy were needed to create the interlaminar delamiaiton on the ZT-CFRP laminates.

This experimental study proved that ZT-CFRP is an effective approach for improving in-plane shear strength and associated strain energy density (i.e., modulus of toughness) due to the reinforcement provided by the CNF z-threads. The ZT-CFRP prepreg is roll-to-roll produced using a modified hot-melt prepregging process; and the ZT-CFRP prepreg can be handled exactly like a regular prepreg to be trimmed, stacked, degassed, molded, and cured into a ZT-CFRP laminate with the traditional OOA-VBO process; these manufacturing characteristics make the ZT-CFRP technology fully compatible with existing composite materials manufacturing infrastructure and can be easily adopted by the users for scaled up applications and large nanostructured CFRP composite parts manufacturing. The findings presented in this study will help the further understanding and development of using long nanotubes or nanofibers to improve the performance of micro-fiber reinforced composites, which can form a possible path toward a next generation of high-performance fiber-reinforced composite materials.

## Supplementary Information

Below is the link to the electronic supplementary material.

Supplementary Material 1

## Data Availability

All data supporting the findings of this study are available within the paper and its Supplementary Information.
